# Large-scale sea ice–Surface temperature variability linked to Atlantic meridional overturning circulation

**DOI:** 10.1371/journal.pone.0290437

**Published:** 2023-08-30

**Authors:** Petru Vaideanu, Christian Stepanek, Mihai Dima, Jule Schrepfer, Fernanda Matos, Monica Ionita, Gerrit Lohmann

**Affiliations:** 1 Alfred Wegener Institute Helmholtz Centre for Polar and Marine Research, Bremerhaven, Germany; 2 Faculty of Physics, University of Bucharest, Bucharest, Romania; 3 Emil Racovita Institute of Speleology, Romanian Academy, Cluj-Napoca, Romania; 4 Faculty of Forestry,” Stefan cel Mare” University of Suceava, Suceava, Romania; 5 MARUM & Department of Environmental Physics, University of Bremen, Bremen, Germany; Woods Hole Oceanographic Institution, UNITED STATES

## Abstract

Due to its involvement in numerous feedbacks, sea ice plays a crucial role not only for polar climate but also at global scale. We analyse state-of-the-art observed, reconstructed, and modelled sea-ice concentration (SIC) together with sea surface temperature (SST) to disentangle the influence of different forcing factors on the variability of these coupled fields. Canonical Correlation Analysis provides distinct pairs of coupled Arctic SIC–Atlantic SST variability which are linked to prominent oceanic and atmospheric modes of variability over the period 1854–2017. The first pair captures the behaviour of the Atlantic meridional overturning circulation (AMOC) while the third and can be associated with the North Atlantic Oscillation (NAO) in a physically consistent manner. The dominant global SIC–Atlantic SST coupled mode highlights the contrast between the responses of Arctic and Antarctic sea ice to changes in AMOC over the 1959–2021 period. Model results indicate that coupled SST–SIC patterns can be associated with changes in ocean circulation. We conclude that a correct representation of AMOC-induced coupled SST–SIC variability in climate models is essential to understand the past, present and future sea-ice evolution.

## Introduction

Variations in sea ice represent a vital indicator of global climate change. The recent drop in the Arctic sea ice, observed in the last decades of satellite monitoring [1–3], has been unprecedented since at least 1850 [4]. This decline results from a combination of anthropogenic forcing [5, 6], and internal variability [7–9], supplemented by additional feedbacks [10–12]. In stark contrast with Arctic sea-ice decline over the satellite era from 1979 onwards the Antarctic sea ice has been slightly expanding [13, 14]. Large disagreement among models is documented regarding the impact of natural variability on Arctic sea-ice evolution during the last 50 years [15, 16]. Furthermore, in sharp contrast with observations, most climate models simulate a significant decrease in Antarctic sea-ice extent over the same period [17–19].

One of the major sources of internal variability within the climate system is the Atlantic meridional overturning circulation (AMOC) [20–23]. It is a major transport pathway for warm tropical waters towards the high latitudes of the Northern Hemisphere. In this manner it redistributes vast quantities of heat and therefore exerts a major control on the planetary energy balance, thus influencing the global climate [24–28]. The AMOC ‘s strength depends on delicate changes in water density and can suffer changes from an active to an inactive state once a certain threshold is reached [29–31]. Some proxy-based reconstructions suggest that the AMOC has been weakening over the 20^th^ Century [32–34]and that its current low amplitude might be unprecedented during the last millennium [35, 36]. However, another proxy-based reconstruction found no significant trend over the last century [37] and the debate is still open on the current state and slowing of the AMOC [38, 39], as well as regarding how much will the AMOC weaken in the near future [40, 41].

Both the AMOC and the Arctic sea ice have been linked to major climate shifts in the distant past [42, 43]. In the, so far relatively stable, current climate, Arctic sea ice variability and the strength of the AMOC are intimately intertwined through a wide range of interactions, including heat transfer mechanisms [44, 45], atmospheric dynamics [26, 46, 47] and ice-albedo feedbacks [48], among others. Previous investigations applying numerical simulations over decades to centuries point to an anticorrelation between the strength of the AMOC and Arctic sea-ice variability [49] and suggest positive feedback that might amplify their relationship [50]. In the 21^st^ century, a weaker AMOC might slow down the pace of the Arctic sea-ice decline by a couple of decades [45]. However, due to uncertainties in the models and lack of long-term observational data, it is not clear how much of the recent Arctic sea-ice changes are due to changes in the AMOC [15].

The main goal of this study is to identify and investigate the impact that changes in the state of the AMOC induce on coupled sea surface temperature (SST)–sea-ice concentration (SIC) variability from 1850 to present. This is achieved using multivariate statistical methods applied on high resolution observed/reconstructed sea-ice concentration data [4], the latest ERA5 Reanalysis product [51], and climate model simulations created using the AWI-ESM2.1 model [52]. In this respect, Section 2 provides a description of the observational data and of the analytical methodology employed in this study. Our main results are presented in the third section. Section 4 presents a discussion of our findings whereas respective conclusions that we draw are provided in the final section.

## Data and methods

### Observational data

The National Snow and Ice Data Center (NSIDC) provides SIC data through the Gridded Monthly Sea Ice dataset [4] extending over the 1850–2017 period, available at https://nsidc.org/data/G10010/versions/2. Information on the state of the sea ice is given as percentage cover at a 0.25°C x 0.25°C spatial resolution. This latest version of sea-ice reconstruction is based on previous NSIDC products [53, 54], and adds improvements on the methodology to combining various sea-ice observational products and applying advanced techniques to estimate sea-ice in areas without any records. The observational products used to generate gridded data are drawn from various sources including historical charts of sea ice around Alaska and Denmark, archives from the Russian Arctic and Antarctic Research Institute, and reports from whaling ships. Since 1979, the main data source is the NSIDC Climate Data Record of Passive Microwave Sea Ice Concentration [2].

SST is provided by the National Oceanic and Atmospheric Administration (NOAA) through the Extended Reconstructed Sea Surface Temperature (ErSST.v5) dataset distributed at a 2°C x 2°C degree resolution and available over the time period from 1854 to 2021. The ErSST.v5 profits from advanced interpolation techniques [55] to create improved gridded observations and is available at https://psl.noaa.gov/data/gridded/data.noaa.ersst.v5.html.

The North Atlantic Oscillation (NAO) index, is obtained as the normalized pressure difference between Gibraltar and Iceland [56] and can be accessed from https://crudata.uea.ac.uk/cru/data/nao. Observations of AMOC changes are from the Rapid Climate Change (RAPID/MOCHA/WBTS) project along 26.5°N [57] that extend from 2004 to 2019. The RAPID AMOC monitoring data is freely available from www.rapid.ac.uk/rapidmoc. Annual mean atmospheric CO_2_ concentration values [58] were obtained from: https://climexp.knmi.nl/start.cgi.

### Reanalysis data

Arctic and Antarctic SIC data are taken from the fifth generation European Centre for Medium-Range Weather Forecasts (ECMWF) reanalysis (ERA5R) [51], that extends over the 1959–2021 period and is available at 0.75° x 0.75° spatial resolution. The ERA5R is the latest reanalysis produced by the ECMWF and provides a variety of atmospheric and climate variables. It is based on a state-of-the-art modelling and data assimilation system that is driven by a large variety of historical observations of pressure, temperature, humidity and other variables. Compared to ERA-Interim, ERA5R is improved regarding parametrization and assimilation technique and could provide a more realistic representation of sea-ice physical processes [54]. ERA5R data are available at https://apps.ecmwf.int/data-catalogues/era5/?class=ea.

From NOAA, we use surface air temperature (SAT) and total precipitation rate (TPR) output from the NOAA-CIRES-DOE Twentieth Century Reanalysis (20CRV3) that extends over the period from 1836 to 2015 [59]. The 20CRv3 is available at https://psl.noaa.gov/data/gridded/data.20thC_ReanV3.html.

### AWI-ESM model

The Alfred Wegener Institute Earth System Model (AWI-ESM, version 2.1) is a state-of-the-art coupled climate model that includes dynamics of land carbon cycle and vegetation [52]. The AWI-ESM2.1 comprises the atmospheric component ECHAM6 [60], that is based on a spectral dynamical core and includes the land surface and carbon cycle model JSBACH [61], as well as the Finite Volume Sea-Ice–Ocean Model FESOM2 [62] that simulates ocean and sea-ice dynamics. The JSBACH simulates the land-based part of the carbon cycle and vegetation dynamics. Earth‘s complex natural vegetation is simplified in the model via plant functional types that may dynamically adjust to, and feedback on, changes in ambient climate [61]. Fluxes of mass, energy, and momentum between ECHAM6/JSBACH and FESOM2 are exchanged between ocean and atmosphere via the OASIS3 coupler. The atmospherere model ECHAM6 is the most recent and final version of the ECHAM family of atmosphere general circulation models developed at the Max Planck Institute for Meteorology (MPI) in Hamburg. The setup that we employ here truncates the series of spherical harmonics in the spectral domain at wave number 63 (T63). In the physical domain our setup employs a Gaussian grid with 47 vertical layers.

The FESOM2 is based on the finite volume approach formulated on unstructured meshes. This numerical method facilitates a very flexible representation of spatial resolution and enables representation of spatially small-scale processes in the global domain while limiting numerical expense. The spatially varying resolution reaches down to 15 km across polar and coastal regions and is in the range of 135 km for the far-field ocean. The link from local dynamics on the global ocean in FESOM2‘s multi-resolution approach has been verified in a number of FESOM-based studies [41, 52, 62]. The AWI-ESM2.1 has been validated for various different climate states including the modern [52], the early-Holocene [63], the Last Interglacial [64], and the Last Glacial Maximum [65]. All modelled quantities presented in this study refer to historical CMIP6 DECK simulations [66], that provides transient climate over the industrial era spanning the period from 1850–2014. The historical simulation was initialized with the equilibrated state derived from the preindustrial simulation. Both simulations follow the CMIP6 protocol [66] and are driven by the respective climate forcing. The historical simulation is forced with observed/reconstructed concentrations of relevant greenhouse, volcanic aerosols and solar forcing. In the preindustrial state the vegetation can evolve freely in the model based on the AWI-ESM‘s implementation of vegetation dynamics. As the historical simulation must represent land cover changes, that occurred during the historical period, precisely in order to create a modelled climate evolution that is as comparable as possible to that derived from observations, dynamic vegetation is deactivated in this simulation and global vegetation cover is instead prescribed as a global time-varying data set.

In this study we focus our analysis on spatially resolved SST and SIC interpolated from the native irregular mesh of the ocean model to a regular grid of 1°x 1° resolution. The modelled overturning circulation is calculated from vertical velocity at the native mesh of the ocean model and the AMOC index is diagnosed as the annual mean time series of meridional volume transport at 26.5°N.

### Multivariate statistical analysis

The dominant modes of Atlantic SST and Arctic SIC variability are obtained by using the method of Empirical Orthogonal Functions (EOF) [67]. This technique applies an orthogonal transformation of a set of observations of correlated variables into a set of values of non-correlated variables. The retrieved uncorrelated variables represent linear combinations of the observed correlated variables. The first main component of the EOF explains the pattern related to the largest variance in the original variables; the second component explains the maximum amount of the remaining variance, and so on. As essentially based on pattern separation, the EOF analysis is an efficient method towards investigating the spatial and temporal variability of of time series of grids.

To identify the coupled SST-SIC patterns we employ Canonical Correlation Analysis (CCA, a multivariate statistical method applied to two fields in order to identify two vector bases [68, 69]. The constraint used by CCA is that the time series associated with each set of basis vectors are maximum correlated. The pairs of patterns (vectors) identified through CCA are ranked in decreasing order of the correlation coefficient of their corresponding time components. If one assumes that distinct forcing factors are characterized by different temporal evolutions, then CCA can be used to separate the footprint of forcing factors on a given field. CCA has been previously used to identify and link coupled SST–sea level pressure [70], coupled SST–global high cloud cover patterns [71, 72] or coupled SST–Drought Severity Index [73] patterns to a specific forcing.

Mathematically, CCA transforms pairs of originally centred vectors X_0_ and Y_0_ into sets of new variables, called canonical variables. The canonical correlations are determined by solving the eigenvalue equations [76]:

$$\left\{ \begin{array}{c} {{[C}_{xx}]}^{-1}[C_{xy}]{{[C}_{yy}]}^{-1}[C_{yx}]W_{x}=\rho^{2}W_{x}\\ {\left[C_{yy}\right]}^{-1}[C_{yx}]{{[C}_{xx}]}^{-1}[C_{xy}]W_{y}=\rho^{2}W_{y} \end{array}\right.$$ (1)

where: C_xx_ and C_yy_ are the matrices of covariance for x and y respectively,

ρ^2^ eigenvalues are the squared canonical correlations, and
W_x_ and W_y_ are the normalized canonical correlation basis vectors.

In order to avoid degeneracy of the autovariance matrix it is recommended to reduce the number of degrees of freedom prior to CCA [69, 74]. This is done through EOF analysis performed using the same number of eigenmodes (EOFs) in each variable. CCA is applied to EOF time series and the canonical correlation patterns are calculated in terms of original variables through linear regression. The main criteria we consider when choosing the number of EOFs is that their cumulated variance is larger than 60% [68]. Theoretically, a higher number of EOFs increases the chances for a better separation. However, after EOF6 the variance explained by each EOF is below 1.5% and therefore they reflect rather noise and do not contribute to an increase of the signal-to-noise ratio. We pre-filter observational SST and SIC data, which include all the physical processes involved in SST/SIC behavior, with the first 6 EOFs. The AWIESM2.1 model outputs are also pre-filtered with the first 6 SST/SIC EOFs. Over the 1950–2021 period, in CCAs performed with ERA5 data, we selected the first 10 SST/SIC EOFs. Similar results are obtained in all CCAs if the number of pairs is increased.

The statistical significance of correlations is examined in relation to the (two-tailed) probability (p-value) to obtain a similar correlation by pure chance. Because significance of correlation of two time series is affected by the autocorrelation of each individual time series, the effective number of degrees of freedom for calculating the p-value is computed with the relation [75]: N_eff_ = N (1—R_1_R_2_)/ (1 + R_1_R_2_), where N is number of values of the time series and R_1_, R_2_ represent the lag-one autocorrelation of each of the two records.

In a preliminary stage, for all datasets used, the annual cycle is calculated relative to the 1980–2010 period and then subtracted in order to analyse and interpret anomalies. Annual means are then computed in order to reduce the number of the degrees of freedom and to increase the signal-to-noise ratio in the data that is used as an input for computation of CCA. Data detrending was performed in order to isolate the forced signal. For SST and SAT data, a linear regression model of the form T = a + b * T_global_ + T’ was fitted at each grid point using the least squares method. T represents either SST or SAT, T_global_ denotes the global mean temperature, and T’ represents the temperature anomaly associated with internal variability. The linear regression enables separation of the forced signal (a+b * T_global_) from the internal variability component (T’). For SIC, the model took the form: SIC = a + b * Index (CO_2_) + SIC’, where Index (CO_2_) represents the CO_2_ forcing signal, and SIC’ signifies the SIC anomaly associated with internal variability or other factors not directly related to CO_2_ forcing. TPR data were detrended in a similar manner.

## Results

### Coupled observed SST–reconstructed Arctic SIC patterns

**Fig 1:**
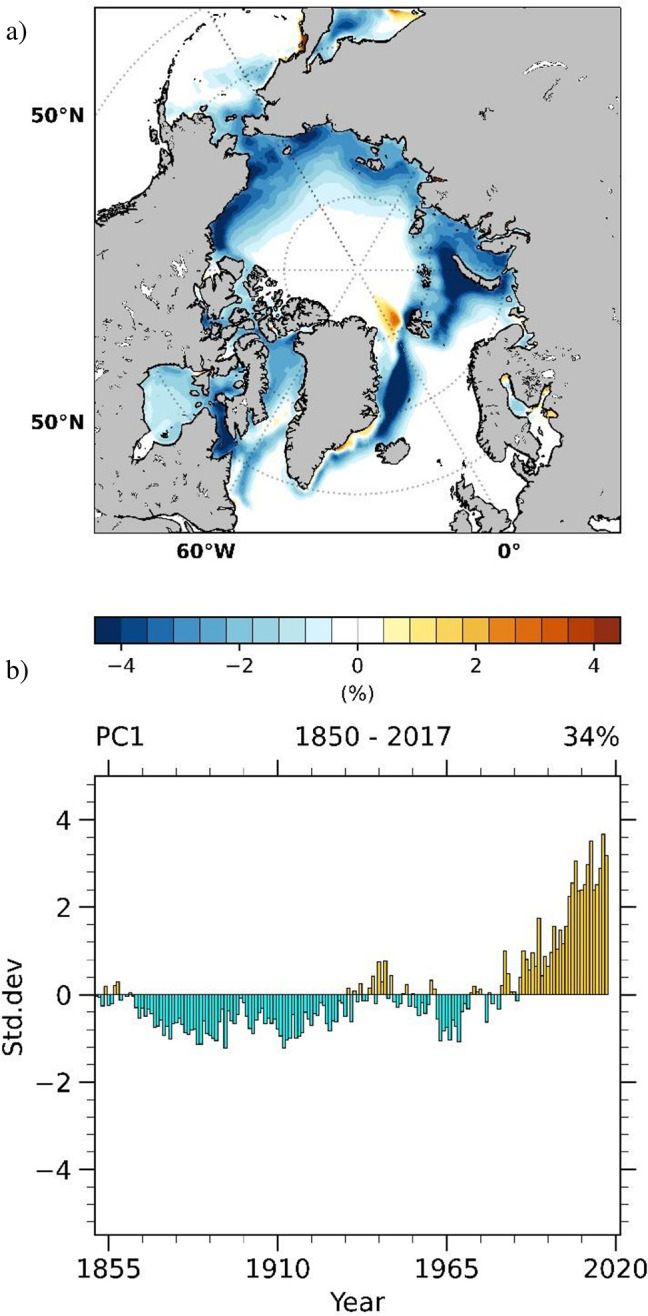
Dominant mode of observed and sea-ice concentration (SIC) variability identified from annual NSIDC SIC anomalies. The pattern of the dominant mode (EOF1) of Arctic SIC variability (a), explaining 34% of variance, together with its associated time series (b).

The spatial pattern of the most prominent mode of SIC variability (EOF1) of observed NSIDC SIC annual anomalies extending over the 1950–2017 period is shown in Fig 1B. It explains ~34% of variance and is dominated by negative anomalies more pronounced over the East Greenland, Barents and Kara Seas and less prominent over Baffin Bay. Its associated time series (Fig 1B) shows an increasing trend, particularly pronounced after the 1980s.

**Fig 2:**
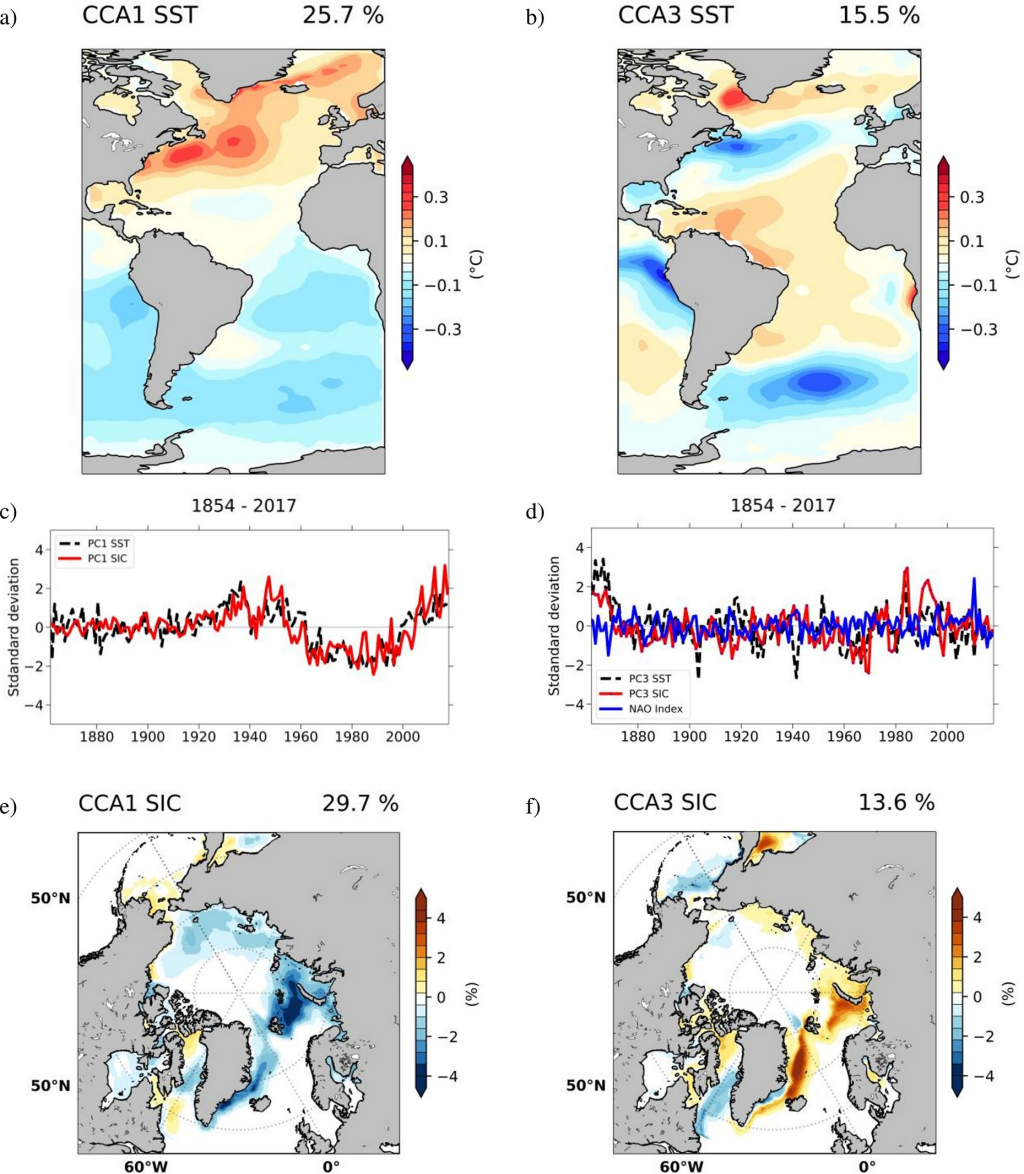
Observed coupled SST-SIC identified between the corresponding ErSST.v5 and NSIDC reconstruction.v2 fields through CCA from 1854–2017. *Left column*: Pattern of SST (°C) (a) from the first pair explaining 26% of variance, and of SIC (%) (e), explaining 30% of variance. Associated time series (c), with SIC (red line), and SST (black line), have a correlation coefficient of 0.72. Right column: Pattern of SST (°C) from the second pair (b), explaining 16% of variance and of SIC (%) (f), explaining 14% of variance. Associated time series (d), with SIC (red line), and SST (black line), have a correlation coefficient of 0.58. Their correlation with the NAO index is 0.48 (0.95 significance).

We investigate possible link between the dominant mode (Fig 1) on the one hand and Atlantic (75°W-15°E,80°S-80°N) SST variability on the other, through CCA between the corresponding annual detrended anomalies from the ErSSTv5 [55] and the latest NSIDC SIC reconstruction [4] extending over the period 1854–2017. Fig 2 highlights the first and the third CCA pair, while the second pair is shown in S1 Fig.

The first pair (Fig 2A) explains 26% of the variance in SST and is characterized by a dipole of SST loadings. Positive anomalies are present in the North Atlantic, these are more prominent over the Gulf Stream and over the north-eastern (NE) coast of Greenland. Negative anomalies prevail over most of the South Atlantic. The Atlantic dipole is a feature previously linked to SST changes due to AMOC variations [22, 23, 25, 28]. In the case of SIC, the pattern of the 1^st^ pair (Fig 2E) explains 30% of the respective variance. It is dominated by negative anomalies to the rims of the Arctic basin and more intense around Greenland, Barents and Kara Seas. This pattern is in good agreement with findings by previous studies that investigate the impact of overturning circulation on Arctic sea ice based on a climate simulation that features slow changes [44] or an abrupt change [45, 46] in the state of the AMOC. Pronounced SIC variability over the Barents Sea has been previously linked to the AMOC through changes in heat transport into the Arctic [76, 77]. It has been shown that AMOC-linked positive temperature anomalies in the North Atlantic can be associated with fluctuations of position and strength of the Aleutian low [25, 78, 79]. This can impact the strength of the poleward winds which then results in the reduction of the sea cover over the Chukchi Sea (Fig 2E). The temporal components of the two spatial structures (Fig 2C) have a correlation coefficient of 0.72 (95% significance) shows a decrease in the mid-60s, followed by a return to positive values in the 2000s. The historical AMOC evolution is uncertain, with disagreements between different types of reconstructions and proxy records [36, 37, 80, 81]. Considering the limitations and uncertainties in proxy-based AMOC reconstructions over the historical period, we argue that it is challenging to directly compare these reconstructions with our time series. Furthermore, here we investigate only the AMOC variability related with coupled SST-SIC fluctuations, but not the whole range of AMOC changes.

To estimate the contribution of the leading CCA pair to the dominant mode of SIC variability, the time series of the leading CCA pair was correlated with the time series of the dominant mode (PC1, Fig 1B). The resulting correlation coefficient is 0.55. The squared correlation coefficient provides an estimate of the proportion of shared variability between two time series [82]. Based on this measure, it can be inferred that approximately 30% (r^2^ = 0.3025) of the variability associated with the dominant mode of Arctic SIC, over the period 1854–2017, can be related to the leading CCA pair.

The SST spatial structure of the second pair, (S1A Fig) explains ~12% of variance and is characterized by centers of opposite signs distributed across the Atlantic Basin. The associated SIC pattern (S1C Fig) explains ~10% of variance. Its maximum loadings over can be found over Baffin Bay, extending towards the Beaufort Sea. Their corresponding time series (S1B Fig) has no significant trend over the analysed period. It shows a small decline until ~1940 and a small increase between 1940 and 2017.

The SST field associated to the 3^rd^ pair (Fig 2B, 16% of Atlantic SST variance explained) features positive loadings over the subpolar gyre and in the region between the equator and 30°N while cold SSTs are observed in the western sub-tropical North Atlantic, just east of the US east coast. This tripolar oceanic response is in part related to the positive phase of the NAO [83, 84] which represents the dominant mode of atmospheric variability in the North Atlantic realm [85]. Its impact on the ocean surface is mainly through changes in turbulent energy flux [84, 86, 87]. The corresponding time components (Fig 2D) are significantly correlated with NAO Index (r = ~048, 95% significance level). The associated sea-ice spatial structure (Fig 2F) explains 14% of Arctic SIC variance and shows positive loadings over the Barents Sea while negative anomalies are observed over Baffin Bay and the Beaufort Sea. The mechanism of the impact of the NAO on SIC across the Barents Sea is via changes in wind anomalies over the eastern Arctic Ocean that increase advection of sea ice out of the Arctic [88, 89]. Over Baffin Bay, in negative phase, the NAO generates negative SIC anomalies by warm air advection into this region [86, 90].

**Fig 3:**
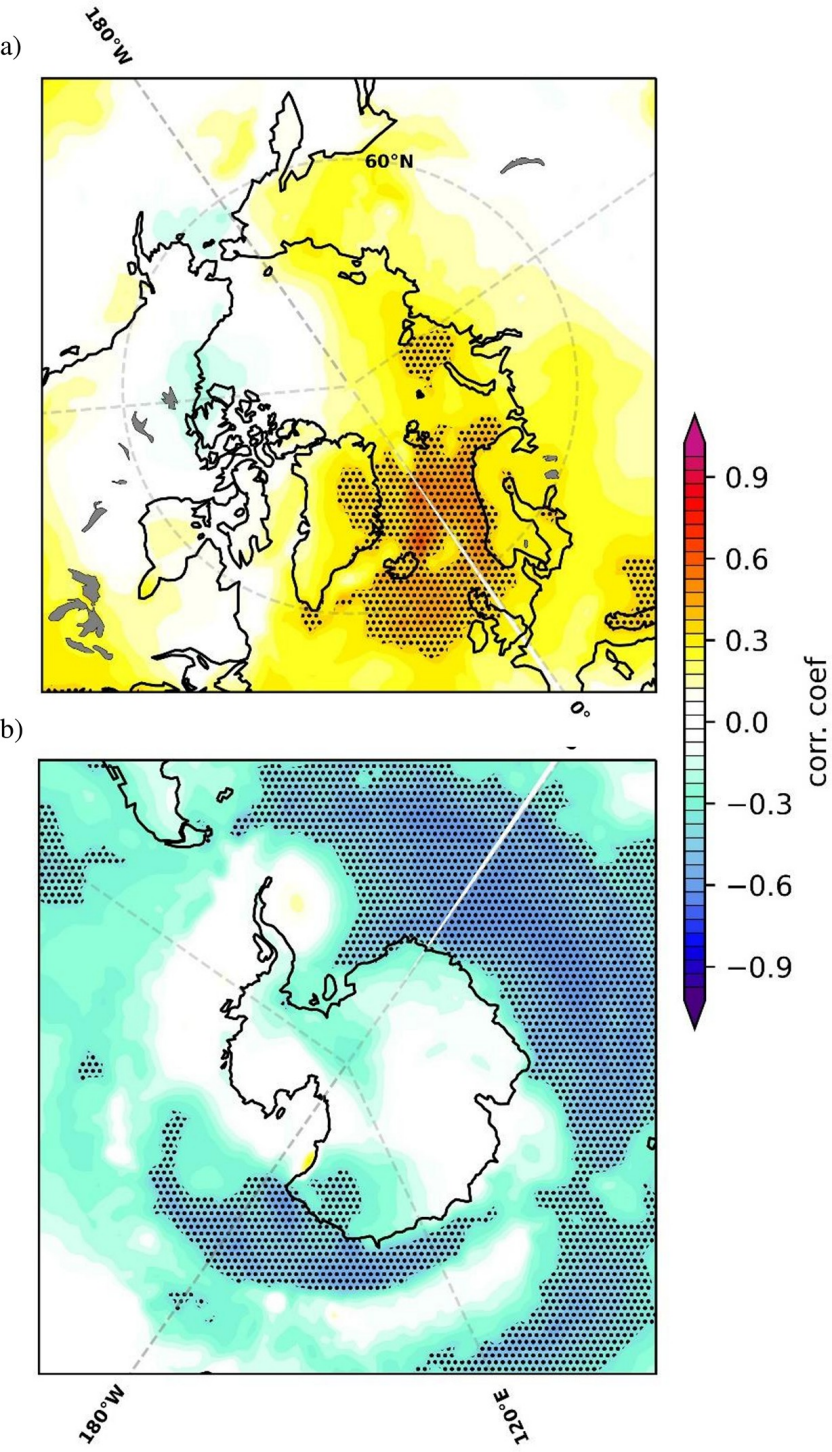
Inter-hemispheric temperature dipole. Correlation map between global detrended annual anomalies from the 20th Century Reanalysis surface air temperature (SAT) and the time series of the observed CCA pair that is investigated in relation to AMOC (Fig 2C), plotted from 55°N– 90°N (a) and from 50°S– 90°S (b), over the 1854–2015 period. The associated statistical significance in hatched areas exceeds 95%.

We test the robustness of the link between the first coupled Atlantic SST–Arctic SIC pair that has been identified through CCA and AMOC. To this end we correlate global fields of surface air temperature (SAT) and total precipitation rate (TPR) of the NCEP/NCAR 20^th^ Century Reanalysis with the time components of the 1^st^ CCA pair (Fig 2C). Fig 3 shows the correlation map of the global SAT field on the time component derived through CCA that is associated with the AMOC. We study their relation across both polar regions, the Arctic (Fig 3A) and the Antarctic (Fig 3B). Hatched areas correspond to a statistical significance level above 95%. Across the Arctic, the highest positive correlation is observed over the Greenland, Kara and Barents Seas (Fig 3A) most likely resulting from changes in northward heat transport induced by AMOC [95]. For the Antarctic we find a significant anti-correlation (Fig 3B). This inter-hemispheric polar dipole in temperature has been previously related to variations in the global energy budget that arise from changes in AMOC [22, 23, 25, 28]. Climate simulations show that the interhemispheric energy imbalance that is induced by the AMOC causes changes in temperature gradients in the Atlantic which in turn shift the position of the Intertropical Convergence Zone (ITCZ) [45, 46]. These features can be distinguished and are statistically significant as interpreted from the TPR correlation map (S2 Fig) that shows a northward shift of the ITCZ in the Atlantic which is in good agreement with the SST map from the 1^st^ CCA pair (Fig 2A).

### Coupled observed Atlantic SST–ERA5 Reanalysis global SIC patterns

**Fig 4:**
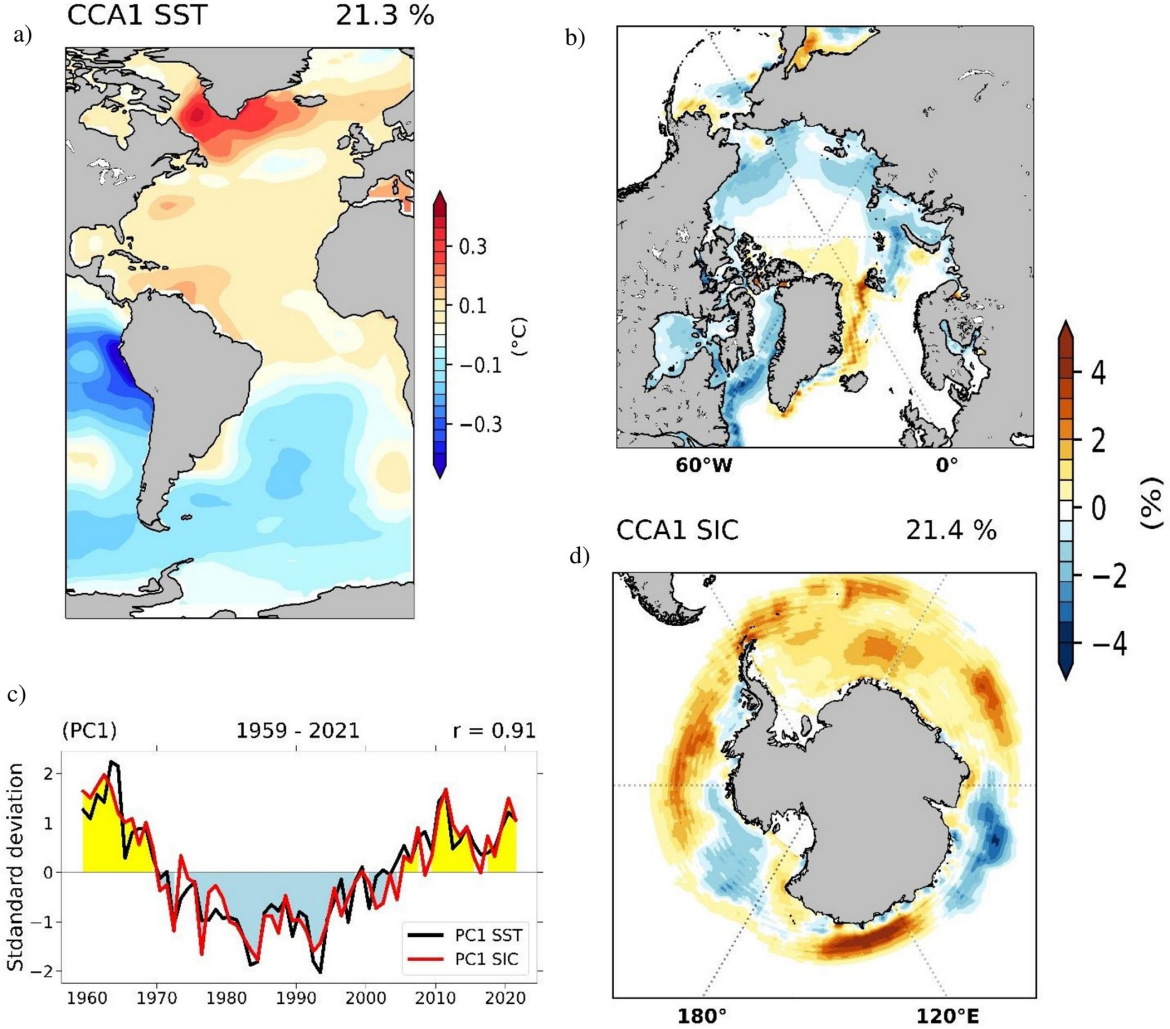
Coupled Atlantic SST—global SIC patterns identified through CCA between the corresponding ErSSTv5 and ERA5 Reanalysis annual detrended anomalies extending over the 1959–2021 period. The SST (°C) pattern (a) explaining 21% of variance and the Arctic (b) and Antarctic (d) SIC (%) spatial structures explaining 21% of variance of the first coupled CCA pair. Their associated time series (c) with SIC (red line), SST (black line) have a correlation coefficient of 0.91.

Spatial structures of SST (Fig 2A) and SAT (Fig 3) show a bipolar response, which implies that changes in Antarctic sea ice are opposed to those in Arctic SIC. If one of them grows, the other shrinks. In order to analyse coupled Atlantic SST–global SIC variability over the period from 1959–2021 we perform a CCA between corresponding annual detrended SST anomalies, taken from from ErSSTv5 [55], and SIC annual detrended anomalies taken from the ERA5 Reanalysis product [51]. The SST structure of the 1^st^ CCA pair (Fig 4A) explains ~21% of the Atlantic SST variance. It can be characterized by uniform negative anomalies in the North Atlantic and loadings of the opposite sign in the South Atlantic, representing the classical multi-decadal SST signature of the AMOC [23, 28, 32]. The associated SIC pattern explains 21% of global SIC variance and shows negative loadings over most of the Arctic region. It is similar in spatial structure with the 1^st^ CCA pair obtained using reconstructed NSIDC Arctic SIC data (Fig 2E), with the exception of the Greenland Sea, where positive SIC values are found. The discrepancy of opposite signs of the two SIC patterns over the Fram Strait can be related to the impact of atmospheric blocking and to local changes in sea-ice export into the North Atlantic [26]. Over the Antarctic (Fig 4D) the SIC spatial structure is dominated by positive loadings extending from the Antarctic Peninsula towards both the Weddell and the Bellingshausen Seas while negative values are observed towards the Pacific Ocean. Since 2004, the RAPID-MOCHA array provides an accurate and reliable measurement of the AMOC [57]. In the time period from 2004 until 2021, the RAPID Index record shows a small decline. This is not observed in the evolution of PC1 until 2010. Over the period 1959–2021 the PC1 from the coupled Atlantic SST–global SIC (Fig 4C) does not show any significant trend.

The identification of a bipolar structure in SST, SAT, and SIC over polar regions supports the hypothesis that variability represented by the previously identified 1^st^ coupled pairs of Atlantic SST–Arctic SIC (Fig 2) and Atlantic SST–global SIC (Fig 4) reflects in large part changes in ocean circulation. These results also indicate that AMOC could represent a forcing factor for the Antarctic SIC trend over the last decades.

### Simulated coupled Atlantic SST—SIC patterns

We aim to further explore the connection between changes in ocean circulation and coupled SST-SIC pairs that we identified in the reconstructed Arctic NSIDC (Fig 2) datasets. To this end we analyse the ocean state from two fully coupled atmosphere-ocean-land surface historical simulations, that have been created with the AWI-ESM2.1 climate model.

**Fig 5:**
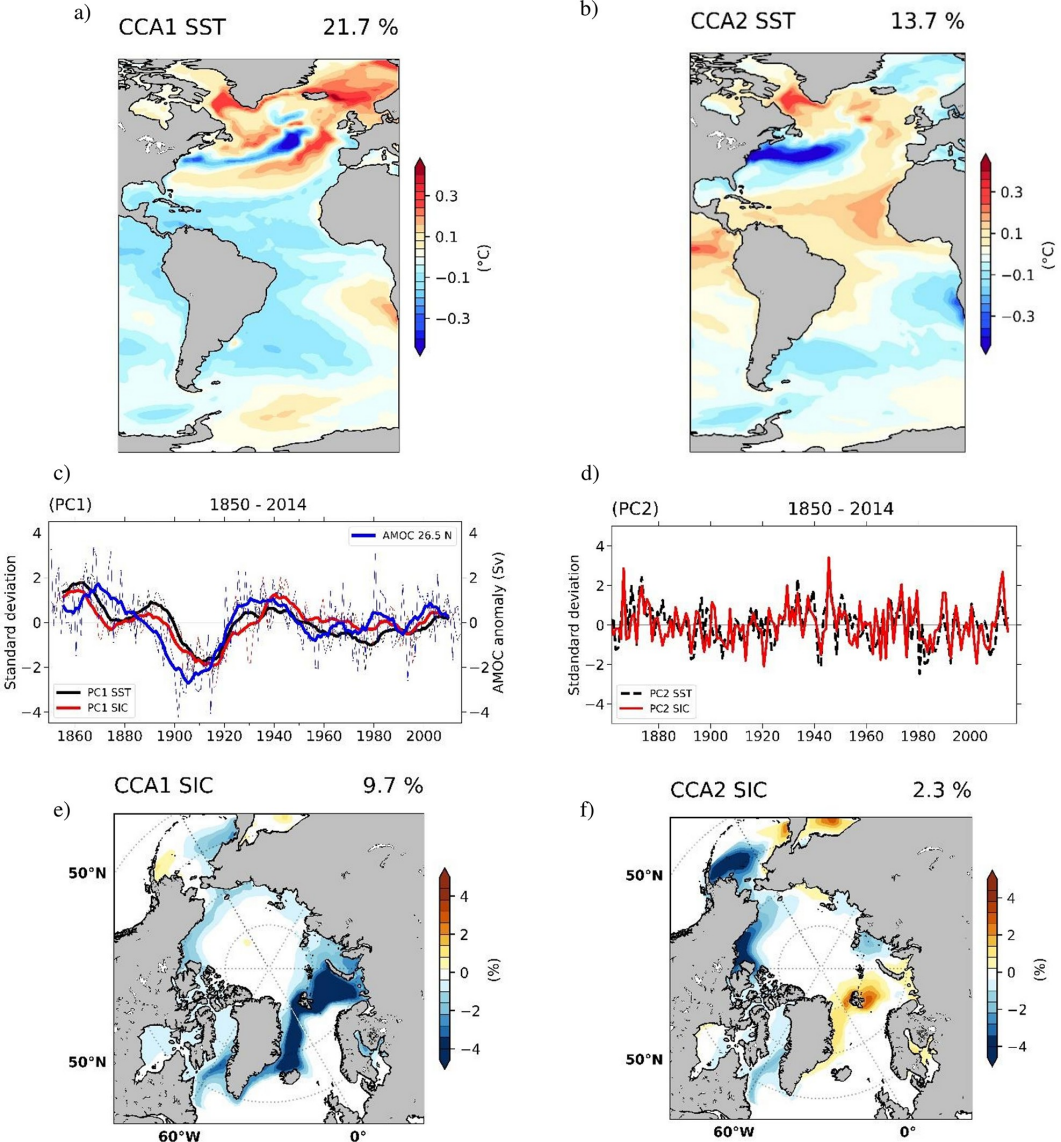
Simulated coupled SST-SIC patterns identified through CCA between the corresponding AWI-ESM-2.1 “historical” annual detrended anomalies from 1850–2014. *Left column*: The SST (°C) pattern (a) of the first pair, explaining 22% of variance and the SIC (%) structure (e), explaining 10% of variance. Their associated time series (c) with SIC (red line), SST (black line) are plotted with an 11yr running mean and have a correlation coefficient of 0.84. Their correlation with the simulated AMOC Index, defined as the time series of annual-mean anomaly of the maximum volume transport streamfunction at 26.5°N (Sv) (blue line) is 0.48 (95% significance level). *Right column*: The SST (°C) pattern of the second pair (b), explaining 14% of variance and the SIC (%) structure (f), explaining 2% of variance. Their associated time series (d), with SIC (red line), SST (black line), have a correlation coefficient of 0.65(95% significance level).

We verify results obtained from reconstructed Arctic SIC data (Fig 2) by performing a CCA of detrended annual average anomalies of Atlantic SST and Arctic SIC derived from the historical simulation over the period 1850–2014. The spatial structure of SST anomalies relating to 1^st^ coupled Atlantic SST–Arctic SIC pair (Fig 5A) features pronouncedly positive anomalies north-east of Greenland as well as negative anomalies over the subpolar gyre and across most of the South Atlantic. The simulated SST pattern explains ~22% of the total variance shows a dipole of anomalies, with positive loadings over most of the North Atlantic and negative values over most of the South Atlantic, similar with the spatial structure of SST anomalies as derived from observations (Fig 2A). An obvious exception are negative anomalies over over most of the central-north Atlantic and the positive loadings over the western coast of Africa and parts of the Southern Ocean. The associated SIC pattern (Fig 5E) explains around a tenth of the total variance in this field and can be described by negative anomalies over most of the Arctic which is in very good agreement with observations (Fig 2C). Temporal evolution of the two structures (Fig 5C) is significantly correlated (r = 0.46, 95% significance level) with the AMOC index derived from the same simulation.

The SST pattern of the second pair (Fig 5B, 14% of variance explained) includes regions of positive anomalies over the subpolar gyre, negative loadings in the western sub-tropical North Atlantic, and warm anomalies in the region between the equator and 30°N. This pattern is similar to the NAO-like SST response that has been identified from observations (Fig 2B). The associated SIC pattern (Fig 5F, 2.3% of total variance explained) includes positive anomalies over the Barents and Kara Seas and negative anomalies over Baffin Bay. It is similar to that of the NAO-linked observed Arctic SIC pattern (Fig 2F), although the percentage of variance explained is significantly lower. The time series of the two spatial patterns (Fig 5D) have a correlation coefficient of 0.56 (95% significance level) and are dominated by inter-annual variability.

The difference between observed and simulated SST spatial structures can be explained by small biases in the AWI-ESM2.1 model, with a cooling one in the North Atlantic and a warming bias in the Southern Ocean and in the South Atlantic coastal upwelling zones [52]. The differences between the observed and simulated SIC structure (not so pronounced over the Chukchi Sea and the Labrador Sea) suggest that the complex Atlantic—Pacific teleconnections are not captured in our model. Despite some discrepancies, the main characteristic of the modelled SST (positive loadings over most of the North Atlantic and negative values over most of the South Atlantic), and SIC (intense negative loadings over the East Greenland, Barents, and Kara seas) are in line with observations (Fig 2). The significant correlation between the time-series of the simulated SST/SIC patterns, that we identified through CCA, and AMOC Index, as simulated in the model, indicates that the 1^st^ observed coupled SST-SIC pair can be interpreted in relation to changes in AMOC.

## Discussion

From satellite-based SIC data sets, we can infer large-scale sea-ice formation and evolution [91]. Yet, owing to satellites being a very recent method of observation of our planet, the related data sets are relatively short compared to reconstructions which consider only indirect evidences [4]. Models can provide as well global and direct inference as satellite observations do, and in principle they can be applied for any arbitrary period of time. On the other hand, models are limited by simplifications and necessary parameterizations of unresolved processes. Finding convergent results using data from NSIDC, ERA5 Reanalysis, and from simulations performed with the AWI-ESM2.1 climate model [55] increases confidence that patterns described and studied here are related to a common mechanism. Through statistical analyses applied on observed SST and reconstructed SIC data over the period 1854–2017 we identify a pair of coupled Atlantic SST–Arctic SIC variability (Fig 2A and 2E). The obtained results indicate that fluctuations in AMOC are affecting the Arctic sea ice through changes in oceanic heat fluxes that modulate the growth/melt of sea ice [24, 44, 49]. The spatial structure of SIC shows the most pronounced anomalies over the East Greenland, Barents, and Kara Seas—i.e. at regions that have experienced the steepest decline in sea ice during the last 50 years [92]. The AMOC can generate the SST structure from Fig 2A through more intense transport of heat towards the pole along the North Atlantic Current, which would explain the SAT heating shown in Fig 3A [93], and just as well the corresponding melting of sea ice over the Greenland, Barents, and Kara Seas that is visible in Fig 2E. A similar SIC response, was identified as a response to changes in the state of AMOC in previous investigations using climate models that simulate a gradual [44, 45] or an abrupt [46] shift in the state of the AMOC and also in some CMIP5/CMIP6 simulations [50].

The subpolar and subtropical branches of the AMOC exhibit distinct characteristics and decadal trends: the subpolar AMOC experienced a buoyancy-forced increase in strength from at least 1980 to the mid-1990s, followed by a weakening over the subsequent two decades, while the subtropical AMOC exhibited a strengthening from 2001 to 2005, followed by a decadal weakening around 2005, with relative stability since the early 2010s [94]. The connection between the two regions is complex, and while some influences may propagate from the subpolar to the subtropical AMOC, the precise mechanisms and timescales of these interactions remain uncertain. We therefore cannot exclude that both branches have an impact on our coupled SST-SIC pair. However, since we find in the model the link between coupled Atlantic SST—Arctic SIC and the AMOC Index defined at 26.5°N, one could argue that our AMOC-linked pairs identified in this study reflect more variation in subtropical sector of AMOC.

Previous studies have shown that a strong AMOC generates warming in the Northern Hemisphere and cooling of the Southern Hemisphere [12, 21, 23, 95]. This inter-hemispheric seesaw is very important for Dansgaard–Oescher events [42] and has been identified in relation to the AMOC during the Holocene [96], although the physical mechanisms of the seesaw are not yet completely understood [95]. In good agreement with the aforementioned studies, the first CCA pair obtained from ERA5 Reanalysis SIC data (Fig 4) shows also a bipolar structure of coupled Atlantic SST–global sea-ice variability: in the Northern Hemisphere, warm Atlantic SSTs [22, 23, 25] are coupled with a decline in Arctic SIC [44, 45]. Likewise, over the Southern Hemisphere negative SST anomalies are coupled with an increase in Antarctic sea ice [97]. This suggests that the Antarctic sea-ice variability over the period 1959–2021 is partly related to variations in the strength of the AMOC. However, we note that on shorter time-scales the Antarctic SIC is affected by additional processes: Rossby waves, that are propagated from the tropical regions of Atlantic and Pacific [98–100], Antarctic Oscillation [101], or changes in the composition of the ozone layer [102]. On the other hand, Arctic sea-ice loss can induce a weakening of the AMOC, through changes in salinity anomalies spreading from the Arctic into the North Atlantic inhibiting deep convection [103]. The weakened AMOC can cause a Southern Hemisphere warming together with a contraction in Antarctic sea ice. This complex relation manifests on centennial time scales, with a lag of ~25–30 years when sea ice leads the AMOC [50] and therefore is not reflected in our analyses.

The AWI-ESM2.1 model is able to reproduce the spatial characteristics of the first coupled Atlantic SST–Arctic SIC pattern identified using reconstructed SIC data. Yet, the model does not show the temporal characteristics of these spatial structures due to internal climate variability [96]. A significant correlation between the strength of the AMOC at 26.5°N and the temporal evolution of the coupled SST–SIC variability, as simulated by the model is also found.

This study presents multiple lines of indirect evidence supporting the association between the AMOC and coupled SST-SIC variability. Firstly, the observed coupled spatial patterns exhibit well-established characteristics associated with the AMOC footprint on Atlantic SST and Arctic SIC. The interhemispheric dipole observed in both SST and SIC spatial structures further strengthens this link. Additionally, the correlation map of SAT aligns with expected AMOC-induced changes and is consistent with the SIC spatial structure. Secondly, the main processes through which AMOC impacts coupled SST-SIC variability, such as poleward heat transport, provide a physically consistent explanation for the observed spatial structures linked with AMOC. Lastly, the link identified in the AWI-ESM2.1 model between coupled SST-SIC pairs displays similar characteristics as in observations, associated with overturning circulation, further supporting our findings, although causality between AMOC and SIC is not irrefutably established.

## Conclusions

Global sea-ice variability is influenced by multiple factors [15]. The coupled Atlantic SST–Arctic SIC spatial patterns are associated with variations in climate modes AMOC and NAO in a consistent manner. Taken together, the two CCA pairs explain a significant amount of variance in coupled Atlantic SST (40%)–Arctic SIC (45%) fields. The AMOC-linked pair can be associated with ~30% of variability in the dominant mode of Arctic SIC during the last ~160 years. A dipolar structure is found in the patterns of the 1^st^ coupled SST—global SIC variability which is linked to AMOC. It explains of roughly a fifth of the variance of global SIC during the last ~70 years, suggesting that AMOC contributes to the Antarctic sea-ice increase observed over recent decades that occurred despite the anthropogenic warming [97, 98]. We propose that the skill of climate models to simulate the rate of future Arctic and Antarctic sea-ice changes shall be tested by quantifying their ability to simulate coupled SST-SIC variability. As AMOC is expected to weaken during the 21^st^ century as a result of increasing anthropogenic greenhouse emissions [34, 35, 104], it is likely that global sea-ice evolution will be influenced by the impact of AMOC changes.

## Supporting information

S1 Fig Observed coupled SST-SIC patterns identified through CCA between the corresponding ErSST.v5 and NSIDC reconstruction.v2 annual fields from 1854–2017. SST (°C) pattern (a) of the third pair explaining 12% of variance, and, the SIC (%) structure (e), explaining 10% of variance. Time series (b), of SIC (red line), and SST (black line) have a correlation coefficient of 0.64.
(TIF)

S2 Fig Correlation map of 20th Century Reanalysis NCEP/NCAR Reanalysis total precipitation rate and the time series of the observed SST/SIC pair associated to AMOC through CCA (Fig 2C) over the 1854–2015 period. The associated statistical significance in the hashed areas exceeds 95%.
(TIF)
