# Supplementary figures and images for: Large-scale sea ice–Surface temperature variability linked to Atlantic meridional overturning circulation
Source: PLoS One. 2023 Aug 30;18(8):e0290437. doi: 10.1371/journal.pone.0290437 (PMC10468057; doi:10.1371/journal.pone.0290437)

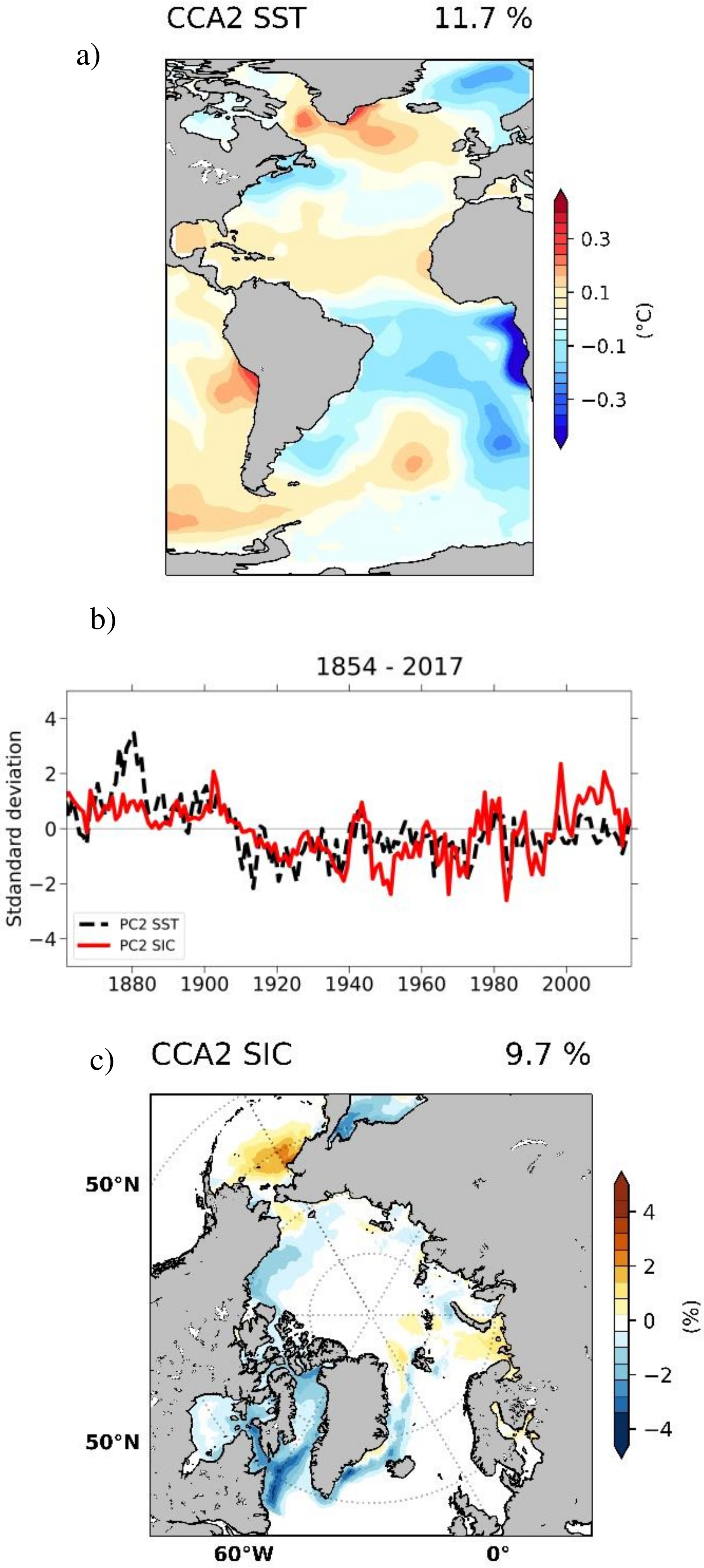

Supplement: S1 Fig — SST (°C) pattern (a) of the third pair explaining 12% of variance, and, the SIC (%) structure (e), explaining 10% of variance. Time series (b), of SIC (red line), and SST (black line) have a correlation coefficient of 0.64. (TIF) [file pone.0290437.s001.tif]

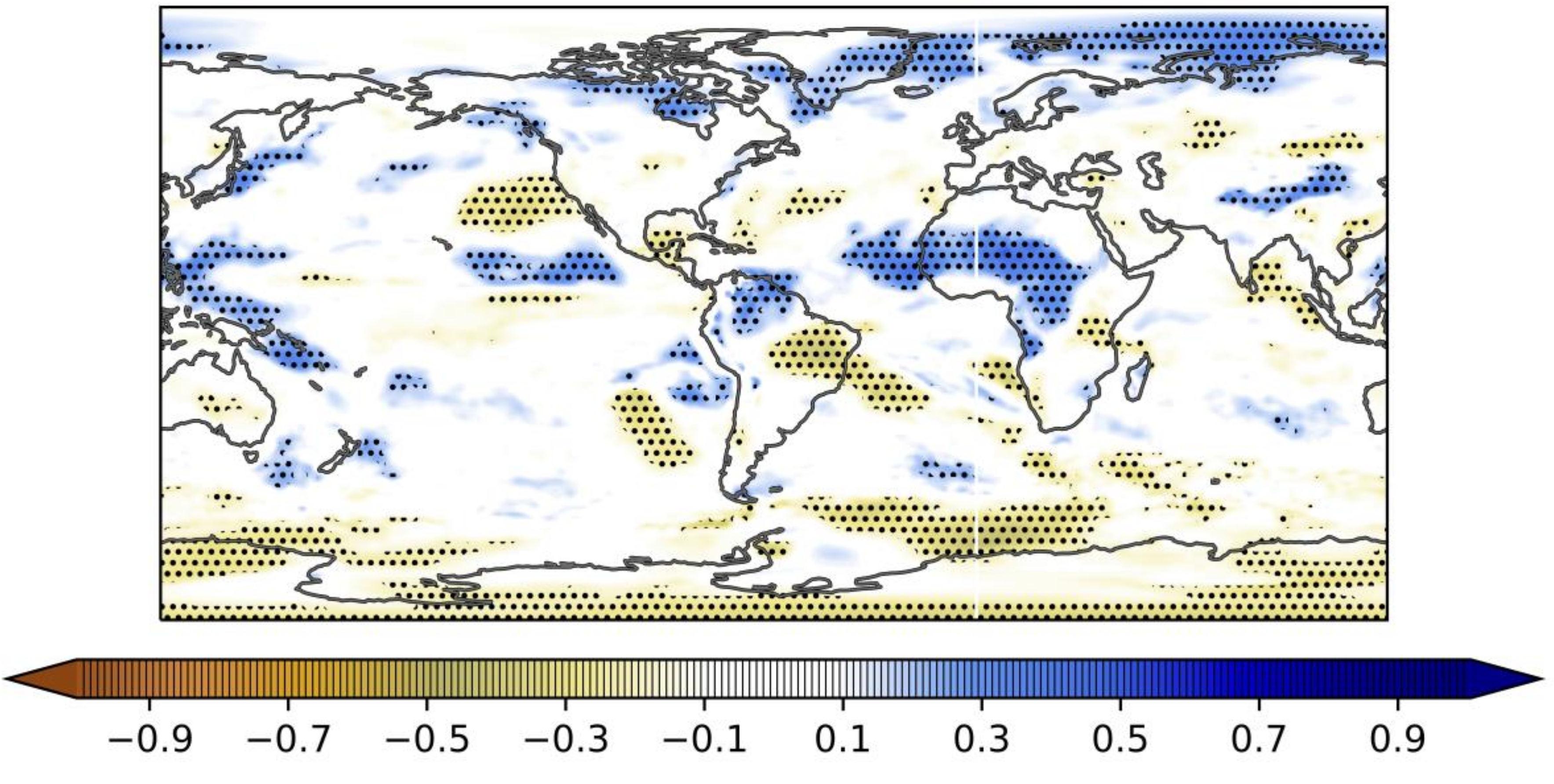

Supplement: S2 Fig — The associated statistical significance in the hashed areas exceeds 95%. (TIF) [file pone.0290437.s002.tif]
